# Memory training and benefits for quality of life in the elderly: A case report

**DOI:** 10.1590/S1980-5764-2016DN1002012

**Published:** 2016

**Authors:** Mariana Medeiros Assed, Martha Kortas Hajjar Veiga de Carvalho, Cristiana Castanho de Almeida Rocca, Antonio de Pádua Serafim

**Affiliations:** 1 Masters Student on the Neurosciences and Behavior Program, Institute of Psychology, University of São Paulo, São Paulo, Brazil.; 2 Collaborating Psychologist at the Neuropsychology Unit, Institute of Psychiatry, University of São Paulo, São Paulo, Brazil.; 3 Psychologist, Head of the Neuropsychology Unit, Institute of Psychiatry, and Collaborating Prof. Dr. of the Department of Psychiatry, University of São Paulo, São Paulo, Brazil.; 4 Director of the Neuropsychology Unit, Institute of Psychiatry, Collaborating Prof. Dr. of the Department of Psychiatry, University of São Paulo, São Paulo, Brazil. Full Prof. of the Post-Graduate Program in Health Psychology, Methodist University of São Paulo, São Bernardo do Campo, Brazil.

**Keywords:** memory and attention training, quality of life, healthy elderly, aging

## Abstract

Studies emphasize the training of cognitive functions to decrease losses in the population. Memory training associated with neurotracker was performed by an 80-year-old man with memory complaints. A battery for measuring memory, quality of life and stress was initially applied and showed low scores. The patient underwent a program for stimulating memory and attention comprising 32 sessions (2 weekly sessions of 90 minutes each). The post-test follow-up showed improvements in the process of storage and retrieval of episodic and working memory, greater use of strategies, faster information processing speed, as well as reduction in complaints and positive impact on quality of life. The results suggest that the use of Neurotracker for training cognitive processes is valid for cognitive rehabilitation programs to promote improvements in quality of life in the elderly.

## RESUMO

Estudos enfatizam o treinamento de funções cognitivas para diminuição de prejuízos na população. Um treino de memória associado ao *neurotracker* foi realizado com homem, 80 anos, com queixas de memória. Foi usada uma bateria para aferição da memória, qualidade de vida e estresse nos quais apresentou baixos escores. Ingressou em programa de estimulação da memória e atenção por 32 sessões (2 sessões semanais de 90 minutos). No pós-teste apresentou melhoras no processo de armazenamento e evocação da memória episódica e de trabalho, maior uso de estratégias, rapidez de processamento de informações, bem como, redução de queixas e repercutindo positivamente na qualidade de vida. Os resultados sugerem que o uso do neurotracker para o treinamento de processos cognitivos se mostra consistentes como programas de reabilitação cognitiva corroborando a melhora da qualidade de vida em idosos.

## INTRODUCTION

Increasing longevity, a common phenomenon in many societies, brings to the fore issues pertaining to health, quality of life and well-being in the elderly population. In this scenario, cognitive rehabilitation programs for stimulating cognitive functions have become commonplace, proving effective in reducing cognitive deficits.^1^ ^,^ ^2^

The study by Balardin^6^ assessed the neural correlates of the efficacy of memory training, using functional magnetic resonance (fMR). The results showed differences in the recruitment of pre-frontal regions in response to the use of coding strategies in specific word coding models between older adults with mild cognitive impairment (MCI) and healthy controls. Initially, performance of the MCI patients was lower than that of healthy controls for retrieval of spontaneously coded information. The increased use of verbal learning strategies associated with greater activation in frontoparietal regions suggests the efficacy of the effects of applying strategies on cognitive mechanisms in both impaired and healthy individuals. 

The study of Barnes et al.,^5^ involving 12 sessions of computer-based cognitive training in older adults with mild cognitive impairment, concluded that the intense mental activity based on cognitive training yielded benefits in individuals with MCI, since a pattern of improvement was observed in learning and verbal memory performance. 

In this context, many computerized techniques have proven useful resources for cognitive training.^1^ ^,^ ^7^ One such tool is Neurotracker,^8^ a computerized program that entails tracking multiple moving targets in a wide 3D field of view. This rapidly stimulates high-level conditions of brain resources essential for working memory, attention and response time processing. This activity recruits various attention systems that are all key components for executive functions, resulting in the stimulation of working memory resources.^8^

Thus, the present study reports the results of a memory training program incorporating the Neurotracker with pre and post-intervention cognitive assessments.

## CASE

We report the case of S.Z., an 80-year-old, right-handed male, married with four children, holder of a Doctorate in architecture and living with family members in São Paulo. There was no history of alcoholism, smoking or pathologies relevant to this report. The patient sought medical assistance in 2014 after presenting frequent memory complaints. Clinical and functional assessments conducted by a neurologist and neuropsychologists revealed no symptoms of cognitive impairment. This result was obtained based on clinical observations and on the Daily Activities Scale and Mini-Mental State Exam,^9^ on which the patient had satisfactory performance for age and educational level, suggesting decline due to the natural aging process. 

The patient was undergoing medical and neurological treatment with the following medications: Galvus Met 850 mg, Betaserc 24 mg, Lipitor (Atorvastatin) 20 mg, Puran T4 50 mg, Rivotril (Clonazepam) 1 mg, Nexum 20 mg, Fluoxetine 20 mg, Benicar 20 mg, and Vitamin D, 8 gts.

In August 2014, the subject was referred for assessment and brain performance training at the Neuropsychology Unit of the Institute of Psychiatry of the Clinicas Hospital of the University of São Paulo School of Medicine. The initial assessment included direct observation of the patient, determination of intelligence quotient, completion of a brief questionnaire on motivation, expectations and impairment prior to training, application of scales measuring self-conception and self-perceived memory, stress, anxiety, depression and quality of life. Tests of attention, memory, information processing speed and reaction time, simple and entailing selection, were also performed. All participants signed the free and informed consent form.

## RESULTS

**Graph 1:**
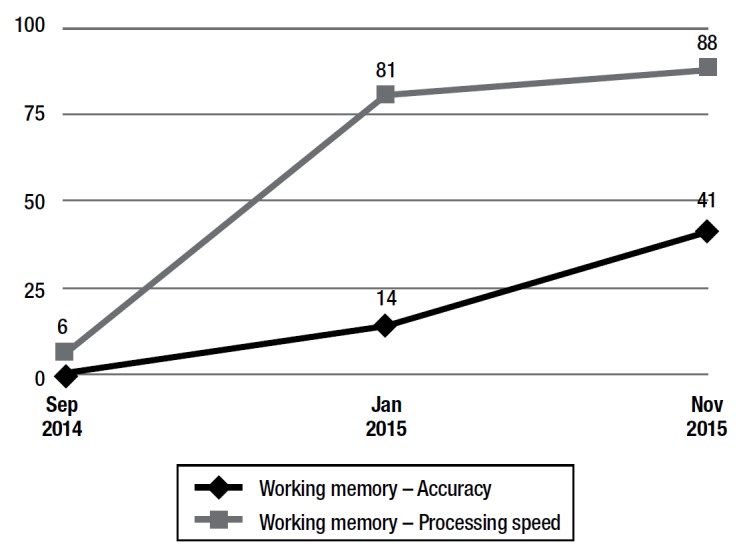
Results of working memory test.

The results of the pre and post-training cognitive assessments are given in both Table 1 and Graph 1.

**Table 1:**
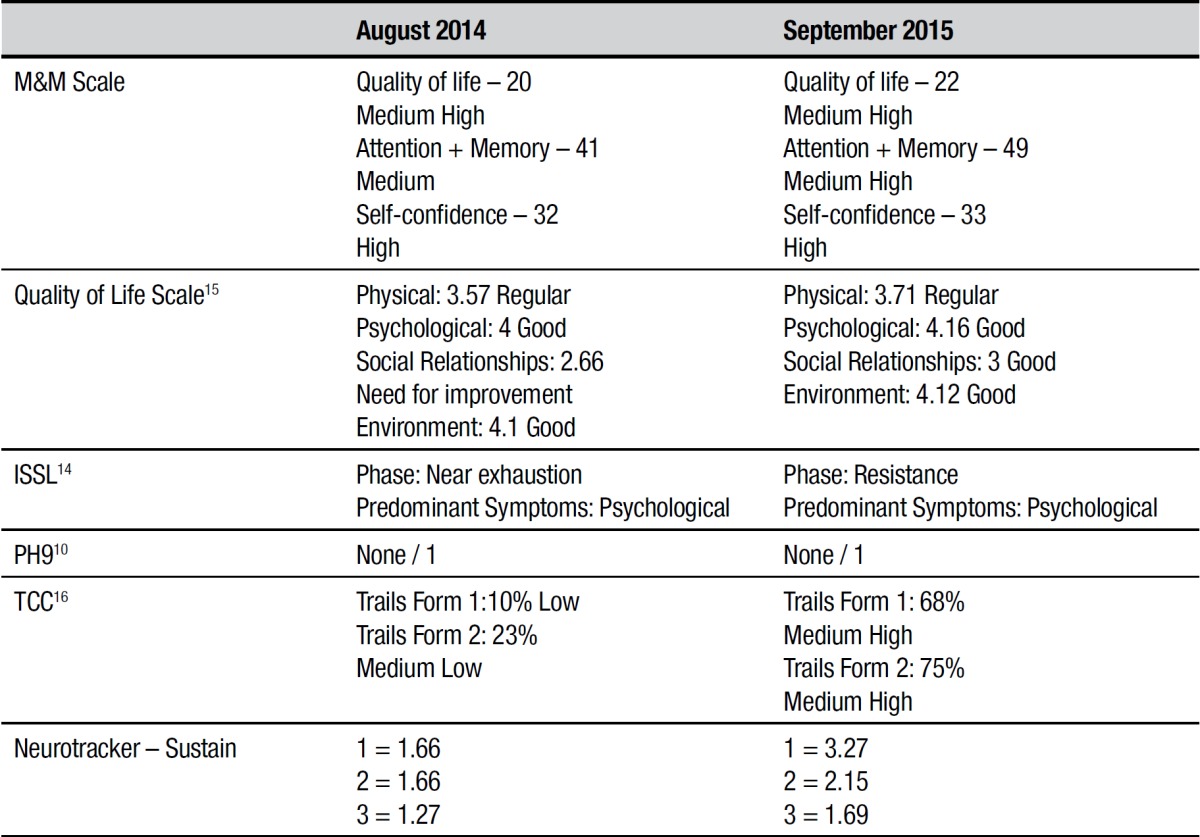
Results pre- and post-memory training.

Pre-training and training. Intellectual performance was assessed using two sub-tests from the Wechsler Abbreviated Scale of Intelligence - WASI,^10^ showing high performance (August 2014) for age and gender. On the M&M Scale, which assesses self-perceived attention, memory, quality of life and self-confidence, the patient was classified as medium high. The rating on attention and memory assessments was medium. The result for self-confidence was high (Table 1).

The subject exhibited no depressive symptoms (Geriatric Depression Scale - Yesavage short form - GDS-15),^11^ but showed stress in the near exhaustion phase with predominant psychological symptoms on the Lipp Inventory of Stress Symptoms for Adults - ISSL.^12^ Results on the quality of life assessment using the WHOQOL-BREF^13^ were for the physical domain: regular; psychological: good; social relationships: need for improvement; and on the environmental domain: good. (Table 1).

The Colored Trails Test^14^ in forms 1 and 2, assessed concentrated, sustained and alternating attention, as well as cognitive flexibility. On the two forms, the patient had a lower-than-expected result for age, educational level and gender (Table 1).

Performance on the Memo Checkup (randomized computerized test for assessing accuracy of episodic and working memory), showed medium episodic memory and significantly lower-than-expected working memory for age (Graph 1). 

Neurotracker^8^ was used as a tool for generating computerized attentional stimuli in Sustain mode - Sustained Attention test; using six trials with one, two and three balls; performed both at pre-training, serving as baseline, and at post-memory training, where stimuli were presented similarly to the attention training protocols.

After the initial assessment battery, the intervention consisting of individual sessions of 90 minutes, twice per week, was run for a 12-month period. The computer was used for the cognitive training, both to access the Neurotracker software and run the Memory Training sessions. The sessions were devised according to the consciously learned mnemonic strategies which required effort to put into practice. The training employed verbal and visual mnemonic methods (image and rhyming cues), expanded repetition (involving presentation of the content to be remembered followed by testing immediately and then later with gradual retention interval), errorless learning (technique for avoiding errors while learning new information), cue removal (stimuli are displayed and gradually withdrawn), PQRST (Preview: preview the material to be retrieved; Question: ask gist questions about the text; Read: read the material carefully; State: state the answers and if necessary reread the text; and Test), association (semantic relationship between words and subsequent retrieval), production (similar to association only sentences must be created for the register) and the categorization process (forming classes of the information to be memorized).^15^

## DISCUSSION

At the post-training stage, improvements were evident in self-perceived attention, memory, quality of life and self-confidence on the M&M Scale. A similar result was found for perceived subjective memory on the MAC-Q scale.^16^

Greater use of the strategy trained was seen on the episodic memory task, suggesting that cognitive training acts as feedback, rendering visible the improvements and consequently, perceptions in terms of well-being and learning strategies, corroborating the results of other studies.^3^

A reduction in stress symptoms was also observed, in line with studies suggesting that cognitive training serves to help improve stress symptoms.^3^ On the reassessment using the WHOQOL-BREF, the results were maintained on physical, psychological and environmental domains (regular, good and good, respectively), but all items were slightly higher on the final score. The social relationships domain improved substantially, whose rating increased to good, corroborating the findings of previous studies.^8^ At post-intervention, the use of learning techniques with mnemonic strategies, reductions in memory complaints and in depressive symptoms,^17^ ^,^ ^18^ as well as increases in processing speed and working memory were evident (Graph 1). This result suggests that the reduction in memory complaints, which possibly contributed to better adaptation to daily activities as perceived by the subject, can mediate stress symptoms.^12^

Training proved effective for sustained and alternating attention, with above-average cognitive flexibility on the Colored Trails test^16^ for patient age, educational level and gender. This finding is relevant in that no references are available in the literature for these test measurements (Table 1).

Results of the computerized memory test showed changes in score during and after training. The results, despite fluctuating at some points, showed a steadily rising curve for both Accuracy and Processing Speed on the test (Graph 1).

Positive results were also obtained on the Neurotracker, the computerized stimuli employed, in the form of a rising curve for scores comparing accuracy, speed and difficulty provided by the software (Table 1).

The results indicate that the use of the Neurotracker for training cognitive processes is valid as part of cognitive rehabilitation programs promoting improvements in the quality of life of the elderly, corroborating the results of previous studies.^1,2,8^

Political issues regarding costs of pension, welfare and healthcare systems oblige societies to seek ways of improving assistance in the aging process. The psychological universe of older adults, besides the biological spectrum (natural or pathological), also encompasses individual potentialities (such as information processing, memory, cognitive performance, among others), in addition to influences from the environment and the sociocultural context.^19^ ^,^ ^20^

Given this scenario, such assistance should include computerized cognitive training programs in private and public institutions for elderly care to improve quality of life and delay the signs of senility during the aging process. Future studies focusing on the development of new techniques and furthering this area should also be encouraged. 
